# Reasons for Refusing Laser-Assisted in Situ Keratomileusis in a Pakistani Population

**DOI:** 10.7759/cureus.1391

**Published:** 2017-06-25

**Authors:** Sharif Hashmani, Nauman Hashmani, Sham Kumar, Sanjay Kumar, Vishal Dhomeja, Sufyan Razak, Hina Rajani, Azfar N Hanfi, Idrees Adhi

**Affiliations:** 1 Ophthalmology, Hashmanis Hospital; 2 Dow Medical College, Civil hospital karachi

**Keywords:** refractive surgery, lasik, laser in-situ keratomileusis

## Abstract

**Purpose:**

To study and analyze the reasons for not performing laser-assisted in-situ keratomileusis (LASIK) surgery in Pakistan.

**Methods:**

This is a retrospective observational review of the patients who presented for LASIK surgery during January 2014 to September 2016 at the Hashmanis Hospital refractive surgery facility in Karachi, Pakistan.

**Results:**

A total of 6005 eyes in 3512 patients presented for LASIK surgery. Out of these, a total of 1795 eyes (29.9%) of 899 patients (25.6%) were rejected. The most common cause for not performing LASIK surgery was found to be increased risk of postoperative ectasia seen in 534 (29.75%) eyes. In 275 (15.32%) eyes, the surgery could not be performed because of affordability of procedure or unscientific apprehensions of the patient. Keratoconus was seen in 268 (14.93%) eyes.

**Conclusion:**

The patients presenting for LASIK surgery need extensive screening as the large proportion of patients may have corneal structural for not performing this procedure. The cost of the procedure plays its role as does the unscientific beliefs amongst the patients.

## Introduction

Laser in situ keratomileusis (LASIK) is among the most popular refractive surgeries in the world [1-2]. It works by creating a flap to lift an intact epithelium and thinning the underlying stroma. The epithelium is then repositioned, without damage; this minimizes pain, inflammation, and the wound-healing response. Previous studies show that this technique is both safe and effective [3-4] with minimal induction of corneal haze [5].

A thorough screening process is required in all patients opting for LASIK to achieve successful and predictable outcomes. Numerous indicators have been highlighted, in the literature, which advises a surgeon whether or not to perform this procedure [6-7]. These include high myopia > 12.00 diopters, keratoconus and a central corneal thickness (CCT) of less than 480 μm. Those considered unfit can then be advised safer alternative procedures, such as a phakic intraocular lens (PIOL) implantation, refractive lens exchange or photorefractive keratectomy (PRK) [6-7].

Although the reasons for not performing LASIK procedure have been documented in the literature [7-10], to our knowledge, no such study has been conducted in Pakistan. This study highlights the reasons for not performing LASIK procedure in our population and the frequency of various refractive errors that were rejected. 

## Materials and methods

This is a retrospective observational review of medical records at the refractive surgery facilities of Hashmanis Hospital in Karachi, Pakistan during January 2014 to September 2016.

All patients underwent the routine examination required which included: uncorrected visual acuity (UCVA), best spectacle-corrected visual acuity (BSCVA), both cycloplegic and subjective refractive error, slit lamp examination, dilated retinal exam, ultrasonic pachymetry, keratometry, and Oculus Pentacam (Pentacam HR; Oculus, Wetzlar, Germany). Approval by the Institutional Review Board of Hashmanis Hospital was obtained and data were collected in accordance with compliance guidelines outlined by the Declaration of Helsinki.

### Selection criteria

Selection criteria used prior to LASIK surgery are listed in Table 1. Other reasons for rejection included any ocular or systemic diseases that are contraindicated, pregnancy, lactation, an unstable refraction or near glasses unacceptable to the patient after presbyopic age. Any patient who did not meet the criteria was not offered LASIK and a maximum of three reasons was included for any single patient. Cataract was detected on a slit lamp examination after mydriasis and cycloplegia. The CCT, on the other hand, was determined by using Oculus Pentacam and was counter-checked by Pocket II ultrasonic pachymeter (Quantel Medical, Inc., Bozeman, MT, USA). Those patients who were unwilling for this surgery did not turn up. Keratoconus was detected clinically and with the help of Oculus Pentacam.

**Table 1:** Table showing the criteria and frequency for rejecting laser-assisted in situ keratomileusis (LASIK) *Spherical Equivalent used for these values, **Value for the entire group in column one Abbreviations: LASIK = Laser in situ Keratomileusis, D = Diopters, CCT = Central Corneal Thickness, RSB = Residual Stromal Bed, BDI = Belin/Ambrosio deviation index, ARTave = Ambrosio’s relational thickness average, ARTmax = Ambrosio’s relational thickness maximum

Criteria		Frequency, n (%)
Age < 18 Years		152 (8.47)
Refractive error (D)	Myopia* < -12	171 (9.53)
	Hyperopia* > 4	52 (2.90)
	Astigmatism > 6.0	7 (0.39)
Corneal parameters	CCT < 480 μm	258 (14.37)
	Presumed RSB < 250 μm	2 (0.11)
Increased risk of postoperative ectasia	BDI < 1.27	534 (29.75)**
	ARTave < 424	
	ARTmax < 339	

The following criterion was used to classify patients into increased risk of postoperative ectasia using the Belin/Ambrosio Display (BAD). Firstly, a deranged Belin/Ambrosio Deviation Index (BAD-D) was used. Secondly, front and back enhanced elevation, thinnest point value, vertical displacement and thickness profile were observed. Any value above 1.27 in any of these parameters was deemed abnormal [11]. Also, we used Ambrosio’s Relational Thickness average (ARTave) and Ambrosio’s Relational Thickness maximum (ARTmax) values. If the ARTave was below 424 and ARTmax was below 339, the eyes were considered abnormal [11].

### Laser-assisted in situ keratomileusis procedure

We used an excimer laser, Alcon EX500 Wavelight (Alcon, Ft Worth, TX, USA), with a 6.0 mm diameter ablation zone and a 1.0 mm transitional zone. The diameter varied from 5.5 to 6.5 mm depending on two factors: Thickness of the cornea and the size of the pupil. LASIK was not performed if the corneal thickness was found inadequate for expected correction with a minimum of 5.5 mm ablation zone.

### Statistical analysis

We used the App sheet to enter data into Google forms. Subsequently, we downloaded the spreadsheet and imported the data into SPSS version 16.0 (SPSS Inc., Chicago, IL, USA). We calculated the frequency and percentage for all categorical variables and the mean, standard deviation and range for all continuous variables. 

## Results

A total of 6005 eyes in 3512 patients presented for LASIK surgery. Out of these, a total of 1795 eyes (29.9%) of 899 patients (25.5%) were rejected; there were 539 females (60%) and 360 males (40%). Mean age of presentation was 27.4 ± 9.02 (1.00 - 70.00) years and as seen in Table 2, a wide range of ages was rejected.

**Table 2:** Table showing the general characteristics Data presented as mean ± standard deviation (range) Abbreviations: Y = Years, M = Male, F = Female, R = Right, L = Left, D = Diopters

Variable	Value
Age (Y)	27.37 ± 9.02 (1.00 - 70.00)
Gender (M/F)	360/539
Eye (R/L)	904/891
Sphere (D)	-4.14 ± 4.84 (-25.00 - 18.00)
Cylinder (D)	-1.69 ± 1.62 (-10.00 - 3.50)
Spherical equivalent (D)	-4.76 ± 4.97 (-26.25 - 17.62)

Table 1 and 3 shows the number and percentage of the reasons for the LASIK operation that were not performed. The most common cause for not performing was found to be increased risk of postoperative ectasia seen in 534 (29.75%) eyes. In 275 (15.32%) eyes the surgery could not be performed because of affordability of the procedure or unscientific apprehensions of the patients about the procedure. Keratoconus was seen in 268 (14.93%) eyes.

**Table 3:** Table showing the other reasons of laser-assisted in situ keratomileusis (LASIK) rejection

Reason	Total	Percentage
Unwilling to pay for surgery	275	15.32%
Keratoconus	268	14.93%
Other	113	6.30%
Forme fruste keratoconus	82	4.57%
Unstable refraction	54	3.01%
Retinal degenerative condition	52	2.90%
Presence of cataract	41	2.28%
Near glasses unacceptable after presbyopic age	30	1.67%
Extreme dry eyes	24	1.34%
Squint	15	0.84%
Pregnancy	9	0.50%
Macular hole	6	0.33%
Glaucoma	4	0.22%
Keratitis	4	0.22%
Nystagmus	4	0.22%
Retinitis pigmentosa	4	0.22%
Macular degeneration	2	0.11%
Uveitis	2	0.11%
Corneal degenerative disease	1	0.06%

**Figure 1:**
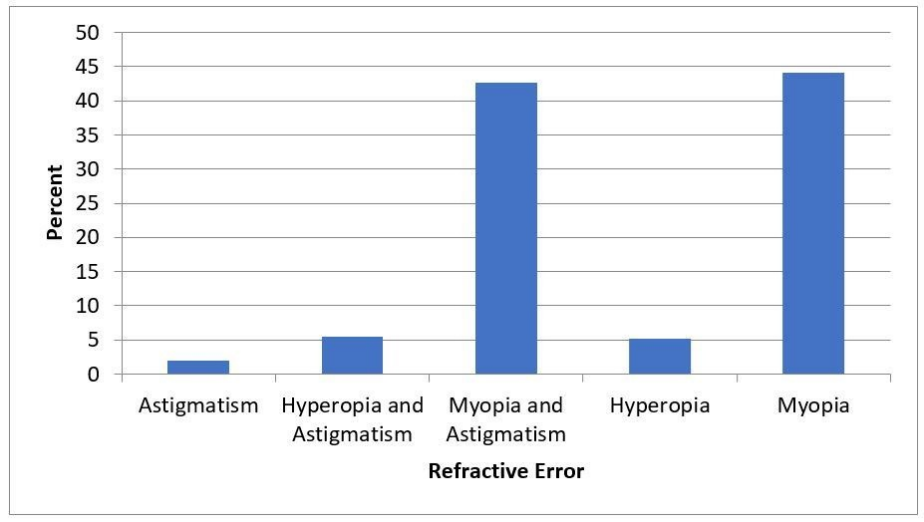
Statistical representation of frequency showing refractive errors Note: Records of 13 patients were not available

Figure 1 shows the most common refractive errors that did not undergo LASIK. The most common refractive errors were either myopia (n=892) or a combination of myopia with astigmatism (n=766) which together accounted for over five-sixths of eyes (86.7%). Our mean spherical equivalent (SE) value was -4.76 ± 4.97 D (range: -26.25 - 17.62). 

## Discussion

Refractive surgeries, like LASIK, are popular in treating those with refractive errors. Although LASIK has been proven safe and effective [3-4], there are certain limitations surgeons have to be wary of [6-7]. When a surgeon fails to identify these problems preoperatively, major side effects can occur which can cause harm to the patient’s vision. Therefore, it is important to identify which factors are most commonly responsible for not performing the LASIK surgery.

In our setup, an increased risk of postoperative ectasia was the most common reason for rejection. Previous studies disagree with us [7-10], two of them found high myopia to be most common while the other two found suboptimal corneal thickness. Latrogenic keratectasia occurs when the cornea becomes too thin due to over ablation. We are particularly careful in noticing any increased risk for this vision-threatening complication due to the limited resources available in the country [12], particularly in ophthalmology. We would rather prefer the patient to be safe than lose his eyesight due to the lack of medical or surgical help.

The second most common reason for not having the LASIK procedure in our study was cost of the procedure and unscientific beliefs about the efficacy of the surgery. This reason has not been highlighted in previous studies. It is understandable that this category is high in our population for two reasons. Firstly, one center of Hashmanis hospital is in a low socioeconomic neighborhood. Secondly, due to the high illiteracy prevalent in this country [12], it is hard to convince patients about the safety of novel procedures.

Our next two reasons, keratoconus and a central corneal thickness (CCT) < 480 μm, correspond to those documented in earlier studies. Both are contraindicated due to postoperative complications like keratectasia [13-14]. Mild or suspected keratoconus, on the other hand, also causes unstable refraction postoperatively [10].

It is important to take into account a patient’s occupation, personality, and mental status before prescribing the surgery. Perfectionists are the hardest to satisfy, due to their extremely high expectations and constant comparison of perfect vision. Such people need to be informed about the various outcomes possible and the associated risks as this procedure does not guarantee perfect vision. Also, we deny patients with this surgery when it has the possibility of yielding inadequate results and, therefore, we have yielded a high satisfaction rate. We recommend other centers to do the same.

We also recommend further research into refractive surgery to cater to a wider audience that wishes to get rid of their refractive errors. We have denied a high variation of refractive errors with myopic and/or astigmatic eyes, being by far the highest number. Other studies agree with the frequency of this refractive error [15]. Currently, our only choices are to offer safer alternatives like intraocular lens implantation, Phakic intraocular lenses or ask them to wait for future innovation.

Our study has several limitations. Firstly, this was a retrospective review and therefore all associated limitations must be considered. Secondly, the study was conducted in the two locations of a private hospital and only a specific social class was observed who were affordable.

## Conclusions

Patients presenting for laser-assisted in-situ keratomileusis (LASIK) surgery need an extensive screening as large proportion of patients may have corneal structural reasons for not performing this procedure. The cost of the procedure plays its role same as the unscientific beliefs amongst the patients.
